# Supplementary material for: CASP microdomain formation requires cross cell wall stabilization of domains and non-cell autonomous action of LOTR1
Source: eLife. 2022 Jan 14;11:e69602. doi: 10.7554/eLife.69602 (PMC8794472; doi:10.7554/eLife.69602)
Supplement: Source data 1. [file elife-69602-supp1.zip › Kolbeck et al_source files/Figure 5-source data 2/Figure 5D_PI uptake copy/20200116_PI uptake complementation lines_full.pdf]

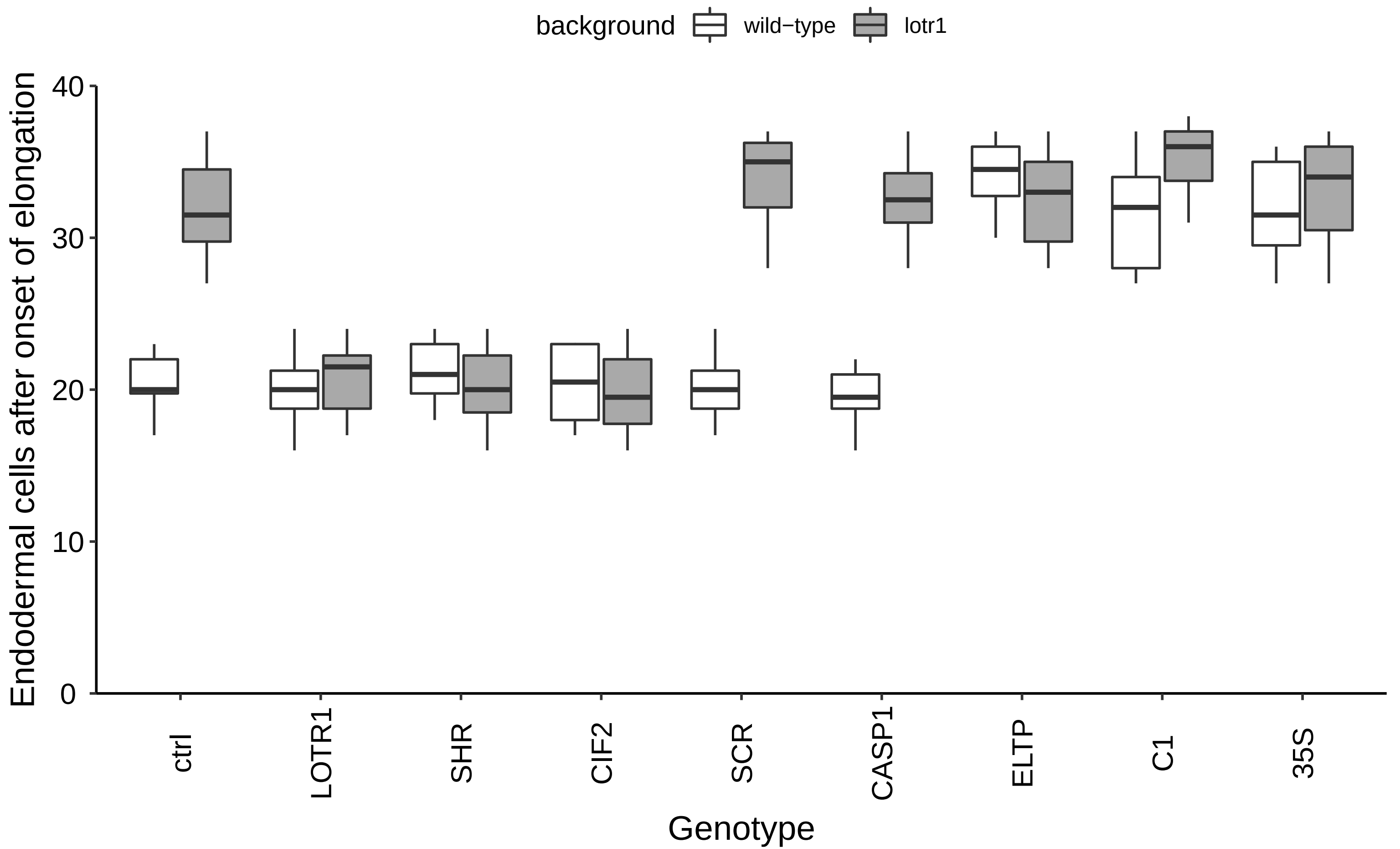

background
wild−type
lotr1
Endodermal cells after onset of elongation
40
30
20
10
0
ctrl
LOTR1
SHR
CIF2
SCR
CASP1
ELTP
C1
35S
Genotype
