# Supplementary material for: CASP microdomain formation requires cross cell wall stabilization of domains and non-cell autonomous action of LOTR1
Source: eLife. 2022 Jan 14;11:e69602. doi: 10.7554/eLife.69602 (PMC8794472; doi:10.7554/eLife.69602)
Supplement: Source data 1. [file elife-69602-supp1.zip › Kolbeck et al_source files/Figure 5-source data 1/Figure 5C_qPCR expression copy/qPCR results tissue specific expression.pdf]

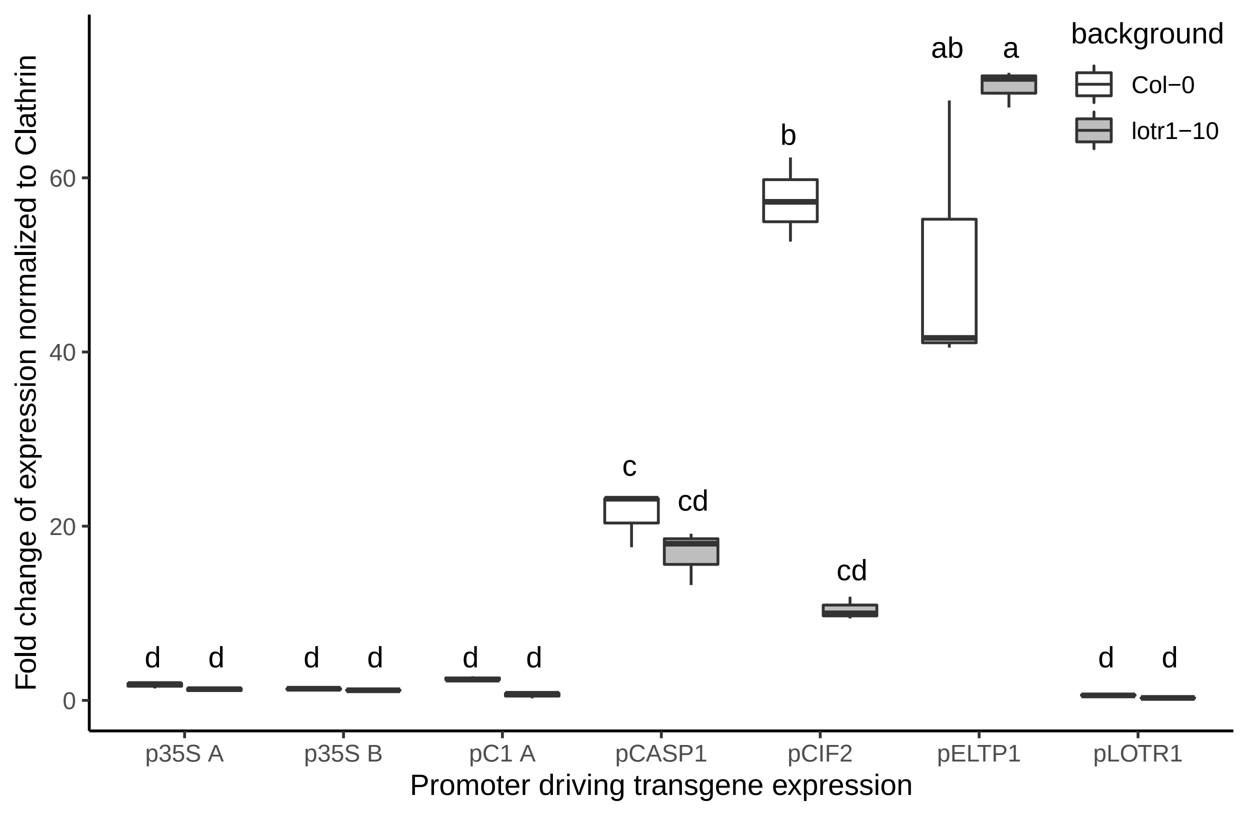

Fold change of expression normalized to Clathrin
0
20
40
60
d
d
d
d
d
d
c
cd
b
cd
ab
a
d
d
p35S A
p35S B
pC1 A
pCASP1
pCIF2
pELTP1
pLOTR1
Promoter driving transgene expression
background
Col−0
lotr1−10
