# Supplementary figures and images for: CASP microdomain formation requires cross cell wall stabilization of domains and non-cell autonomous action of LOTR1
Source: eLife. 2022 Jan 14;11:e69602. doi: 10.7554/eLife.69602 (PMC8794472; doi:10.7554/eLife.69602)

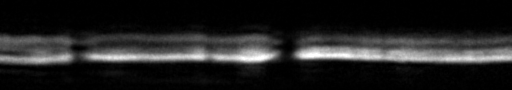

Supplement: Source data 1. [file elife-69602-supp1.zip › Kolbeck et al_source files/Figure 2-source data 3/Figure 2G_membrane coordination correlation copy/sgn1_crop.tif]

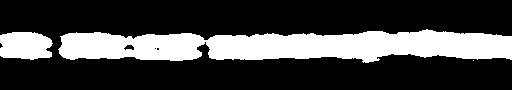

Supplement: Source data 1. [file elife-69602-supp1.zip › Kolbeck et al_source files/Figure 2-source data 3/Figure 2G_membrane coordination correlation copy/esb1_crop_bin.tif]

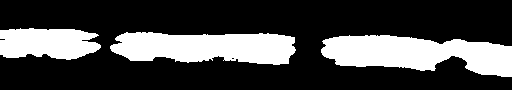

Supplement: Source data 1. [file elife-69602-supp1.zip › Kolbeck et al_source files/Figure 2-source data 3/Figure 2G_membrane coordination correlation copy/sgn3_crop_bin.tif]

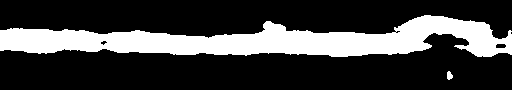

Supplement: Source data 1. [file elife-69602-supp1.zip › Kolbeck et al_source files/Figure 2-source data 3/Figure 2G_membrane coordination correlation copy/lotr1_crop_bin.tif]

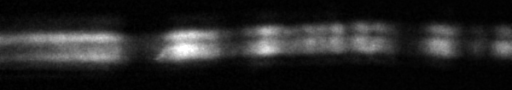

Supplement: Source data 1. [file elife-69602-supp1.zip › Kolbeck et al_source files/Figure 2-source data 3/Figure 2G_membrane coordination correlation copy/CASP-GFP_crop.tif]

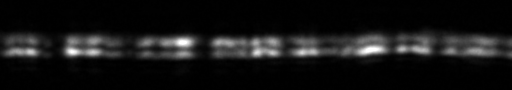

Supplement: Source data 1. [file elife-69602-supp1.zip › Kolbeck et al_source files/Figure 2-source data 3/Figure 2G_membrane coordination correlation copy/esb1_crop.tif]

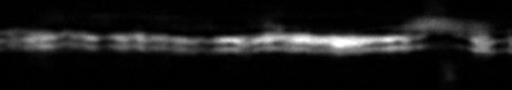

Supplement: Source data 1. [file elife-69602-supp1.zip › Kolbeck et al_source files/Figure 2-source data 3/Figure 2G_membrane coordination correlation copy/lotr1_crop.tif]

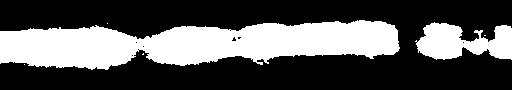

Supplement: Source data 1. [file elife-69602-supp1.zip › Kolbeck et al_source files/Figure 2-source data 3/Figure 2G_membrane coordination correlation copy/CASP-GFP_crop_bin.tif]

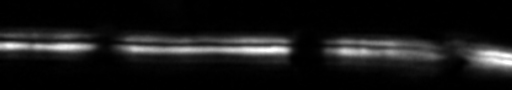

Supplement: Source data 1. [file elife-69602-supp1.zip › Kolbeck et al_source files/Figure 2-source data 3/Figure 2G_membrane coordination correlation copy/sgn3_crop.tif]

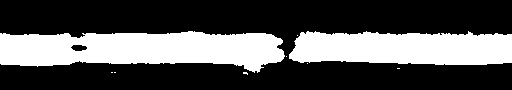

Supplement: Source data 1. [file elife-69602-supp1.zip › Kolbeck et al_source files/Figure 2-source data 3/Figure 2G_membrane coordination correlation copy/sgn1_crop_bin.tif]

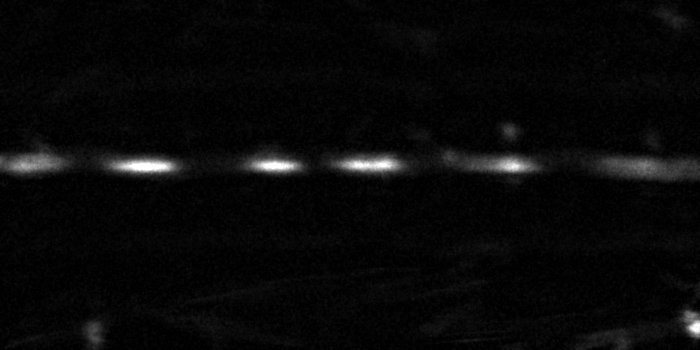

Supplement: Source data 1. [file elife-69602-supp1.zip › Kolbeck et al_source files/Figure 2-source data 2/Figure 2E,F_ablation membrane quantification_young copy/Young_pre_Ch13_fil_bg.tif]

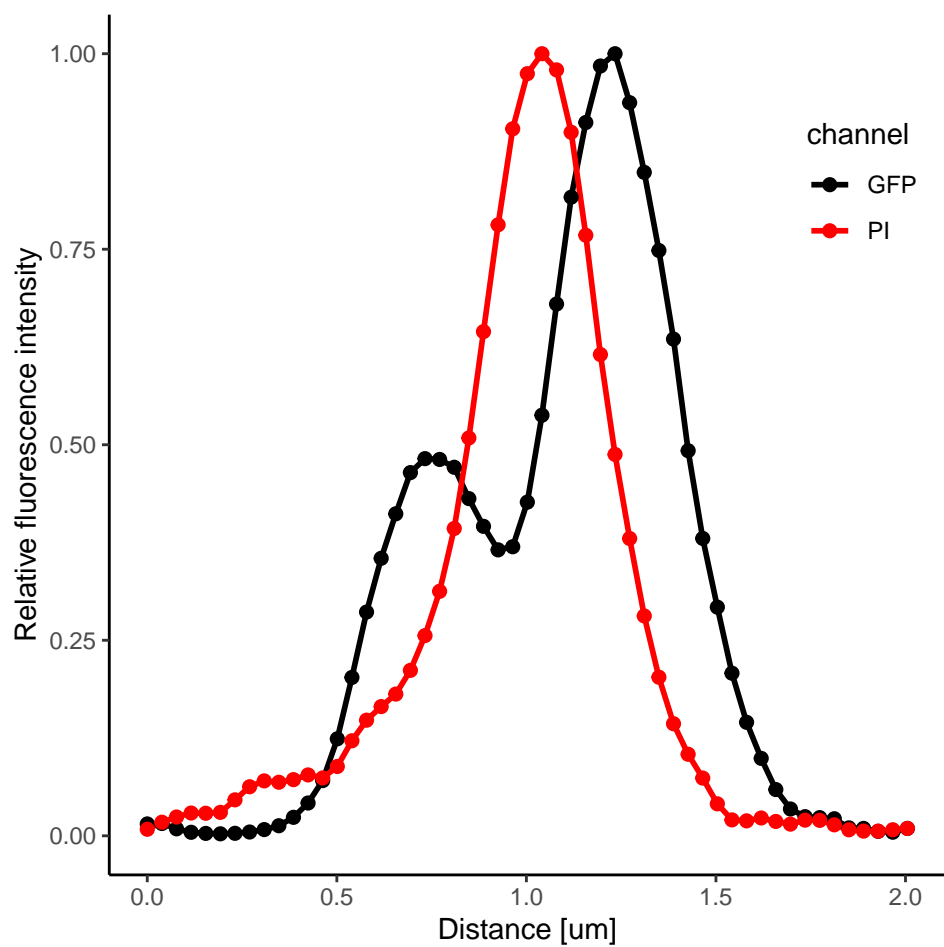

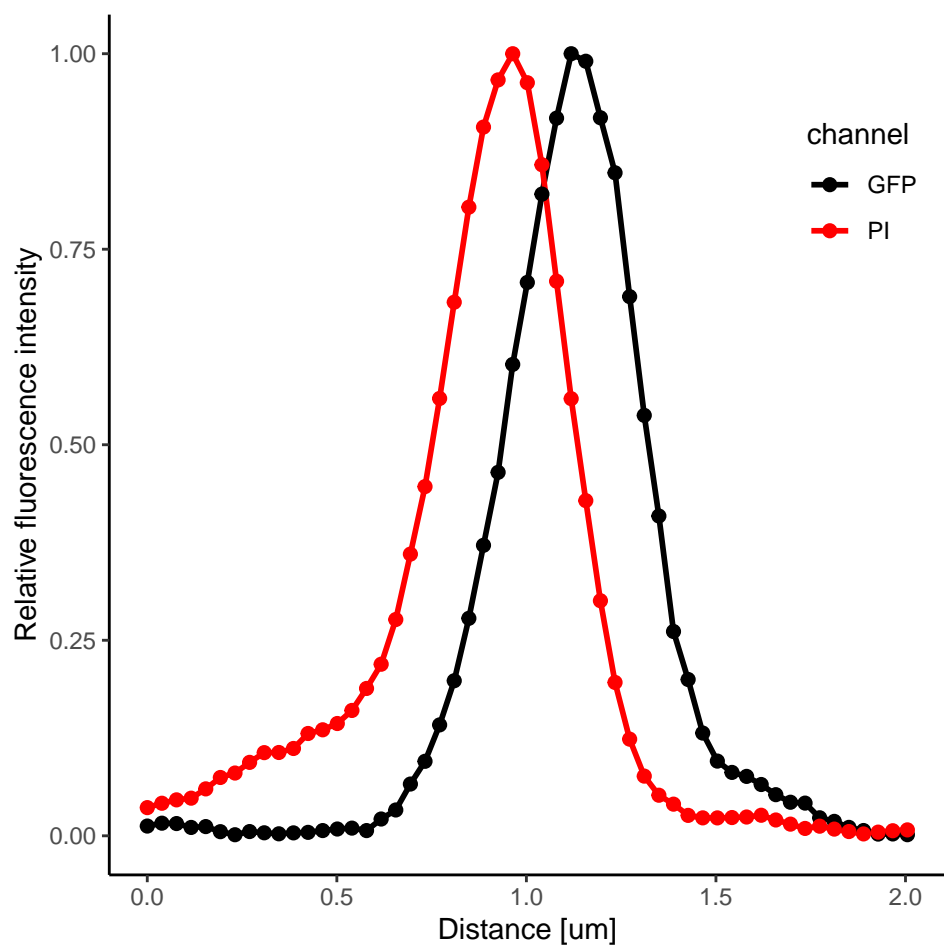

Supplement: Source data 1. [file elife-69602-supp1.zip › Kolbeck et al_source files/Figure 2-source data 2/Figure 2E,F_ablation membrane quantification_young copy/Ablation_intensity_young.pdf]

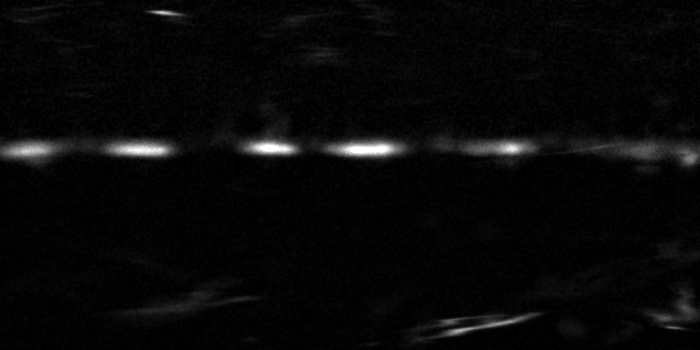

Supplement: Source data 1. [file elife-69602-supp1.zip › Kolbeck et al_source files/Figure 2-source data 2/Figure 2E,F_ablation membrane quantification_young copy/Young_post_Ch13_fil_bg.tif]

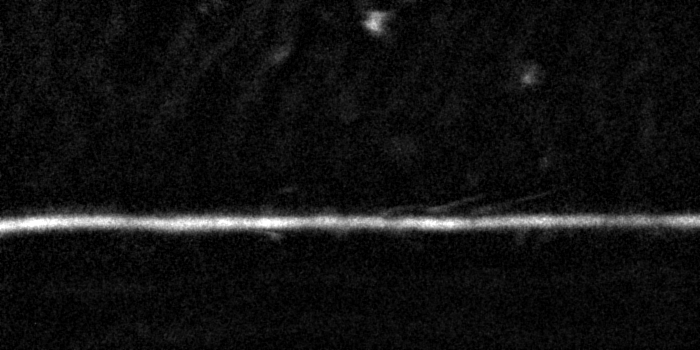

Supplement: Source data 1. [file elife-69602-supp1.zip › Kolbeck et al_source files/Figure 2-figure supplement 1-source data 1/Figure 2-figure supplement 1C,D-source data 1_ablation membrane quantification_mature/Mature_pre_Ch13_fil_bg.tif]

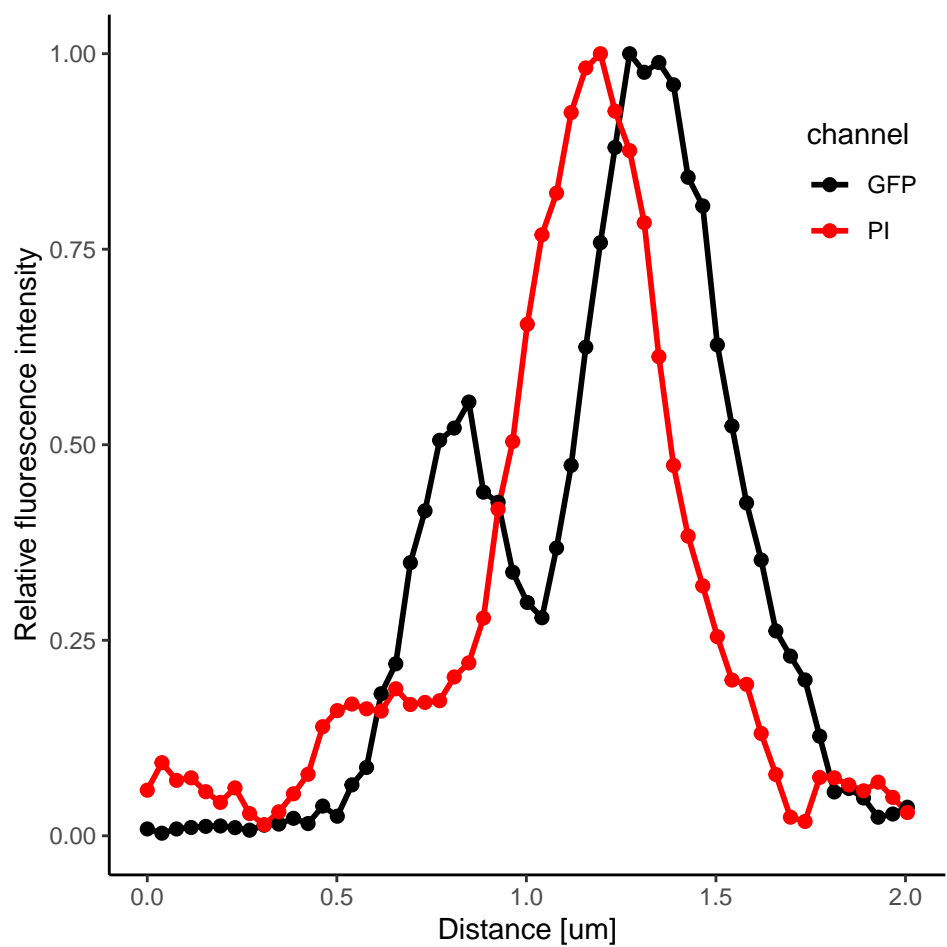

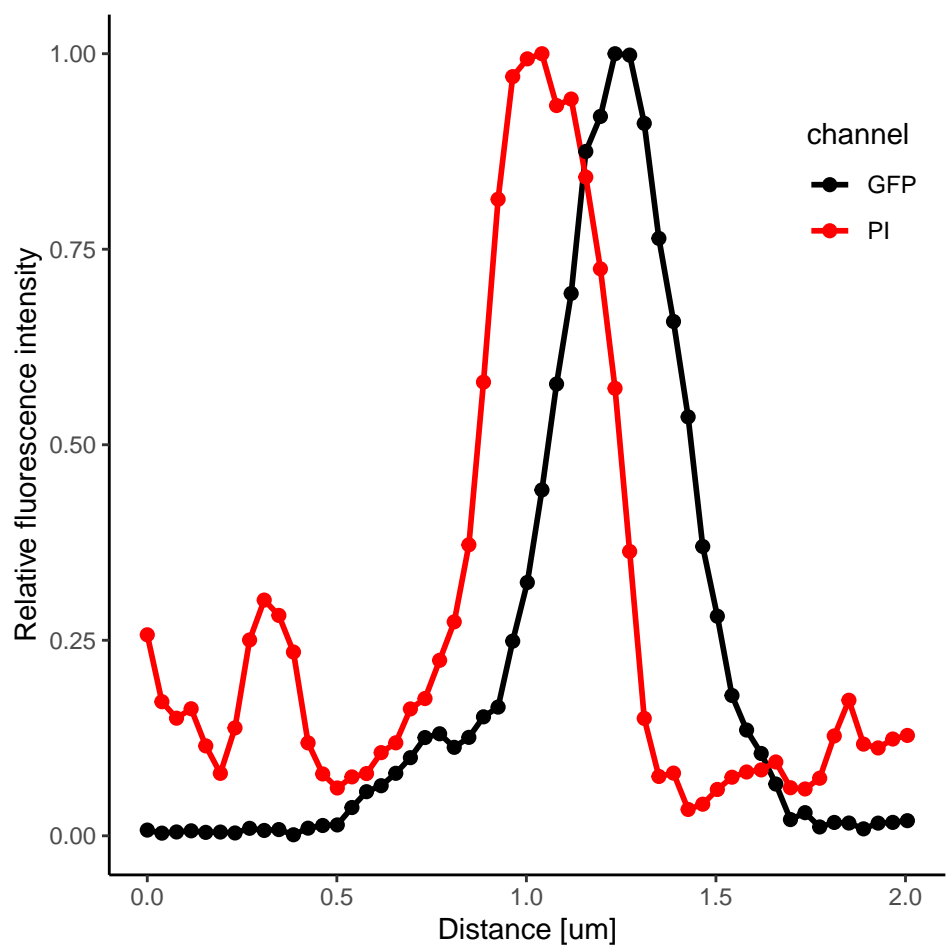

Supplement: Source data 1. [file elife-69602-supp1.zip › Kolbeck et al_source files/Figure 2-figure supplement 1-source data 1/Figure 2-figure supplement 1C,D-source data 1_ablation membrane quantification_mature/Ablation_intensity_old.pdf]

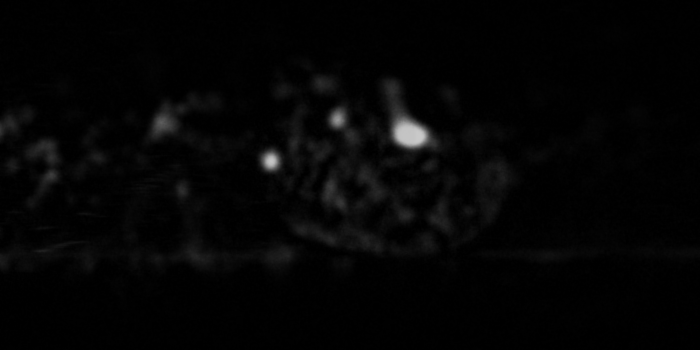

Supplement: Source data 1. [file elife-69602-supp1.zip › Kolbeck et al_source files/Figure 2-figure supplement 1-source data 1/Figure 2-figure supplement 1C,D-source data 1_ablation membrane quantification_mature/Mature_post_Ch13_fil_bg.tif]

Ectopic CASP1-GFP deposits [EN-PE/total]

a

1.00  
0.75  
0.50  
0.25  
0.00

lotr1-10  
Genotype

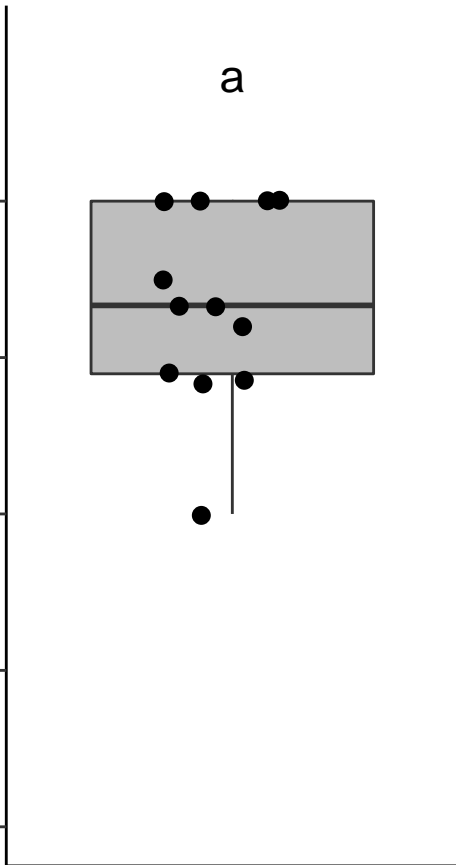

Supplement: Source data 1. [file elife-69602-supp1.zip › Kolbeck et al_source files/Figure 1-figure supplement 1-source data 1/Figure 1-figure supplement 1B_patch distribution/Patch distribution.pdf]

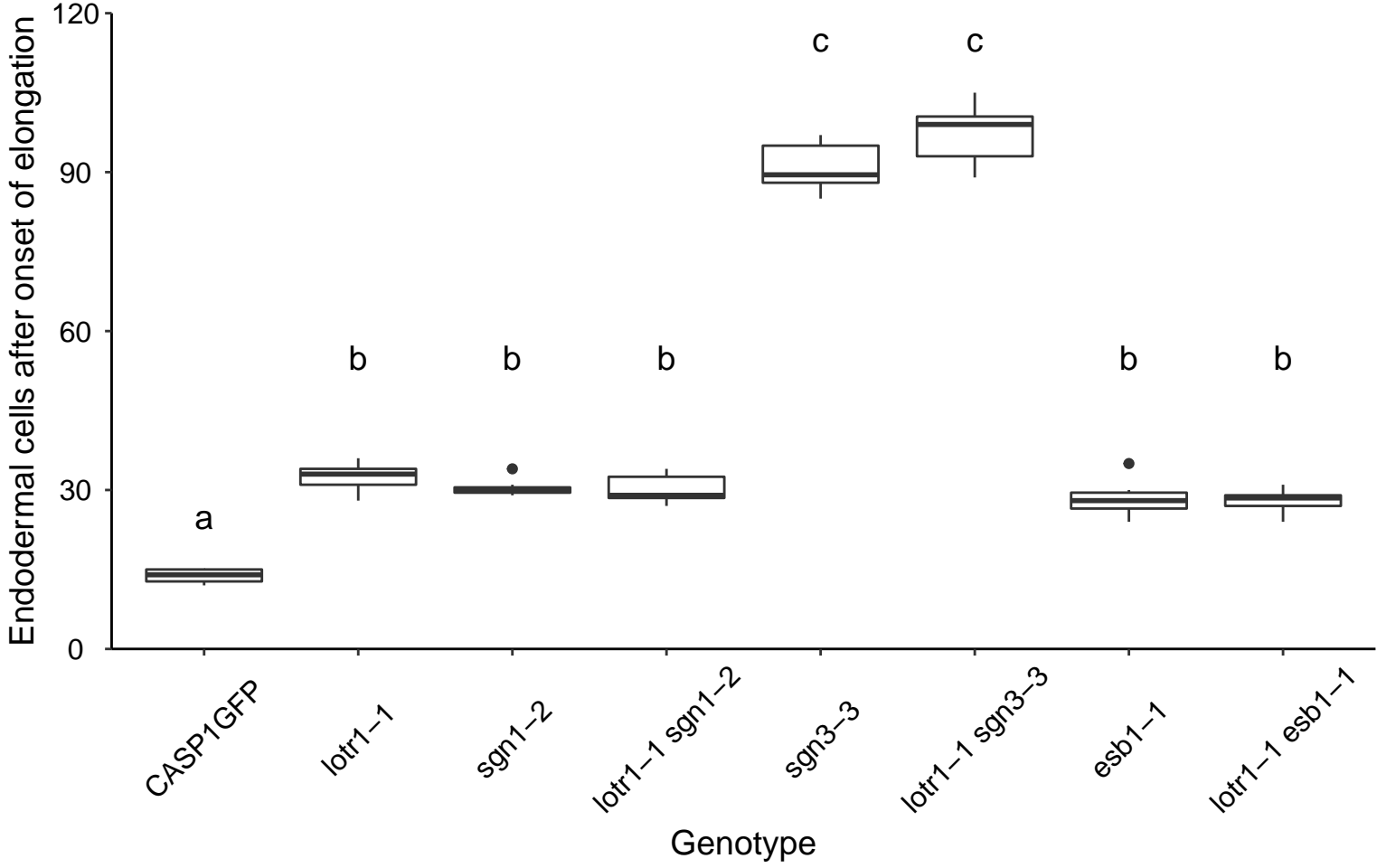

Supplement: Source data 1. [file elife-69602-supp1.zip › Kolbeck et al_source files/Figure 4-source data 1/Figure 4C_PI uptake copy/20170314_PI uptake mutants.pdf]

—●— ablation —●—

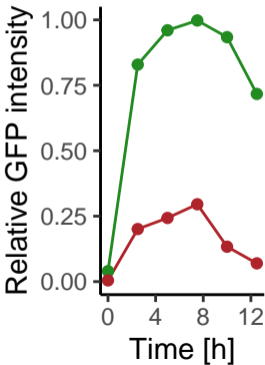

Supplement: Source data 1. [file elife-69602-supp1.zip › Kolbeck et al_source files/Figure 3-source data 1/Figure 3B_neighbour ablation copy/Ablation_intensity.pdf]

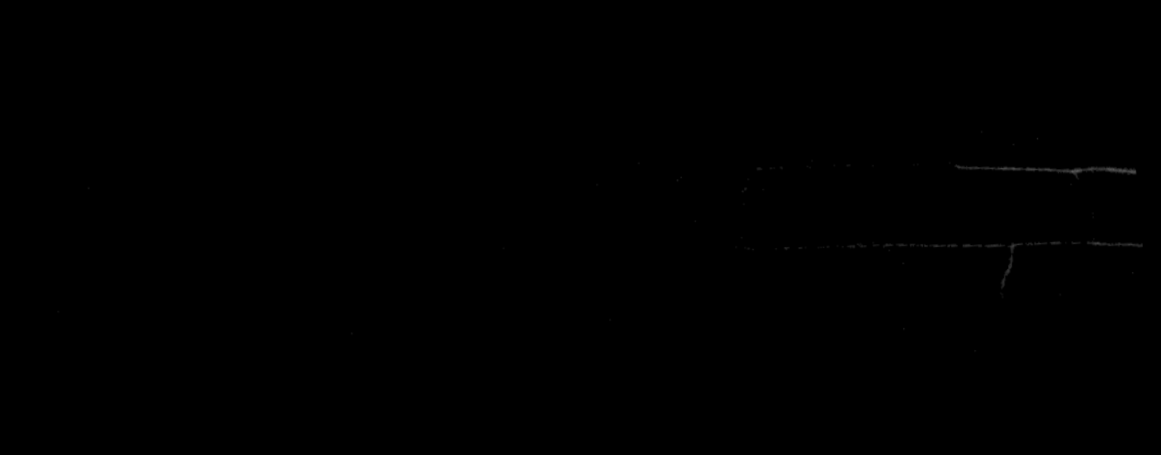

Supplement: Source data 1. [file elife-69602-supp1.zip › Kolbeck et al_source files/Figure 3-source data 1/Figure 3B_neighbour ablation copy/Stack_reg.tif]

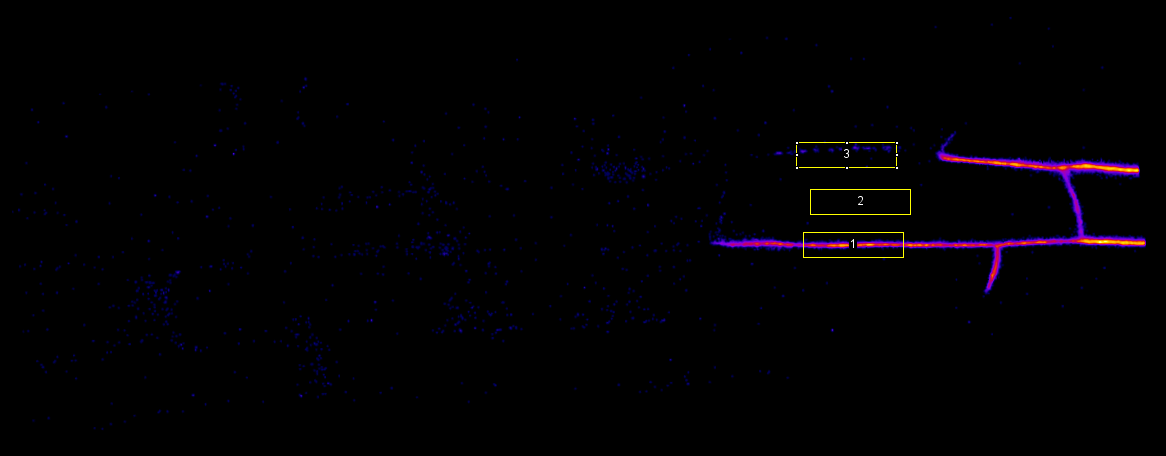

Supplement: Source data 1. [file elife-69602-supp1.zip › Kolbeck et al_source files/Figure 3-source data 1/Figure 3B_neighbour ablation copy/Roi_overlay.tif]

Relative GFP intensity to the mean

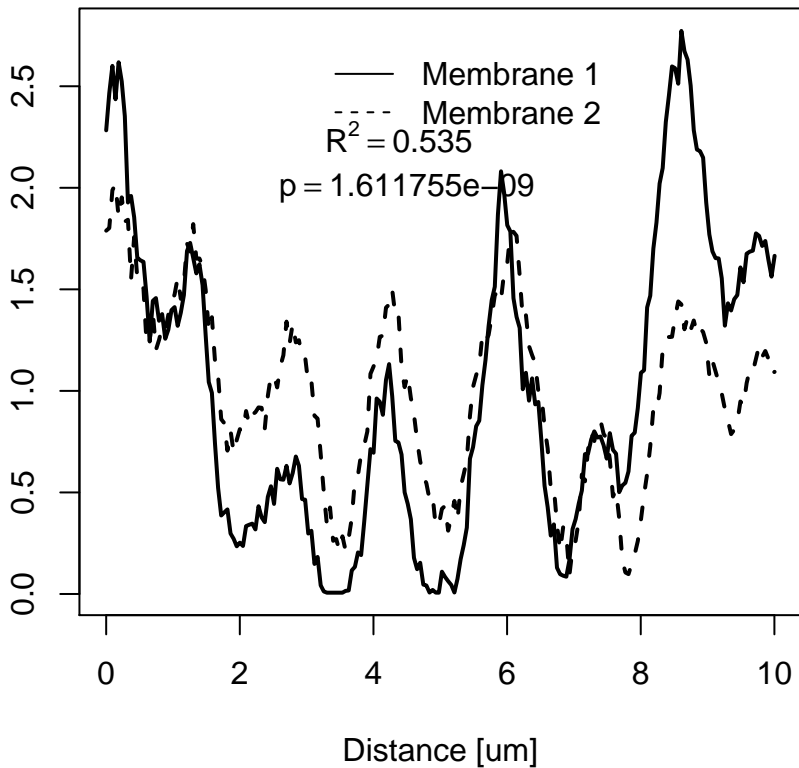

Supplement: Source data 1. [file elife-69602-supp1.zip › Kolbeck et al_source files/Figure 2-source data 1/Figure 2B_double membrane coordination copy/GFP intensity coordination.pdf]

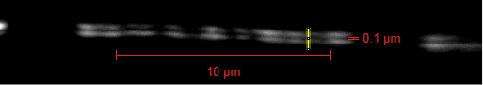

Supplement: Source data 1. [file elife-69602-supp1.zip › Kolbeck et al_source files/Figure 2-source data 1/Figure 2B_double membrane coordination copy/CASP1-GFP_annotated.tif]

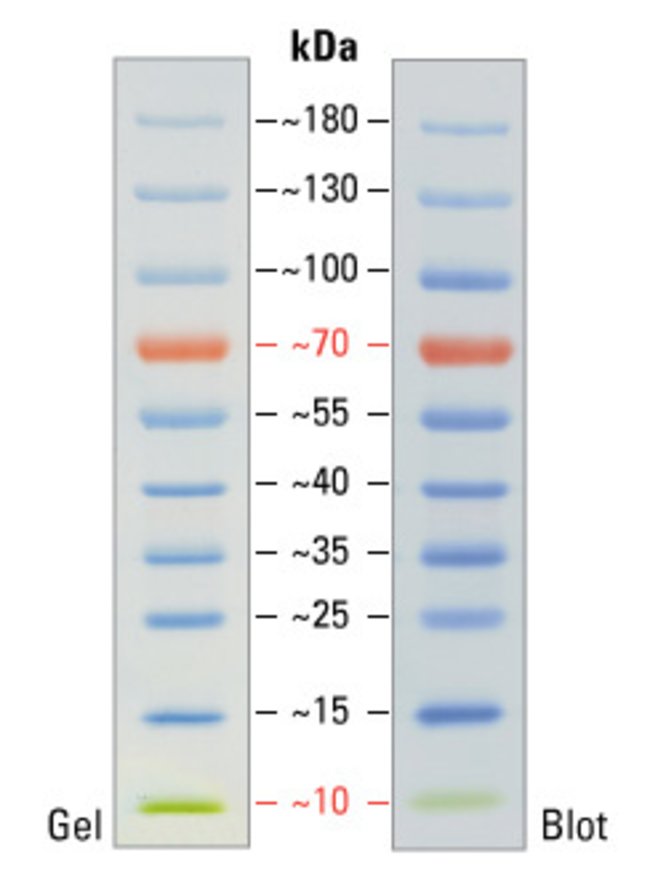

Supplement: Source data 1. [file elife-69602-supp1.zip › Kolbeck et al_source files/Figure 4-figure supplement 1-source data 1/Figure 4-figure supplement 1C-source data 1/PageRuler pre-stained ladder.jpg]

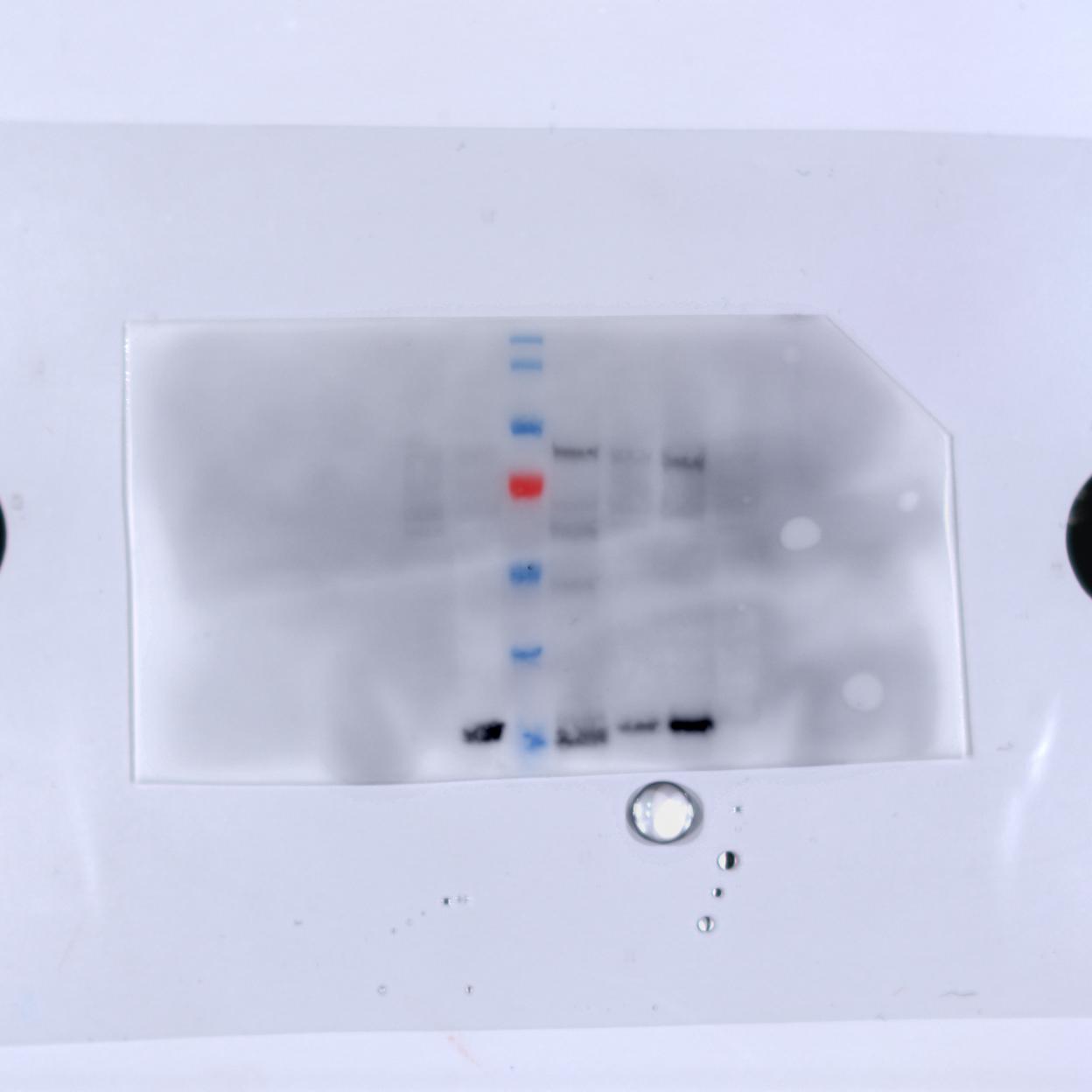

Supplement: Source data 1. [file elife-69602-supp1.zip › Kolbeck et al_source files/Figure 4-figure supplement 1-source data 1/Figure 4-figure supplement 1C-source data 1/RFP 20190404_125027_Ch+Marker.jpg]

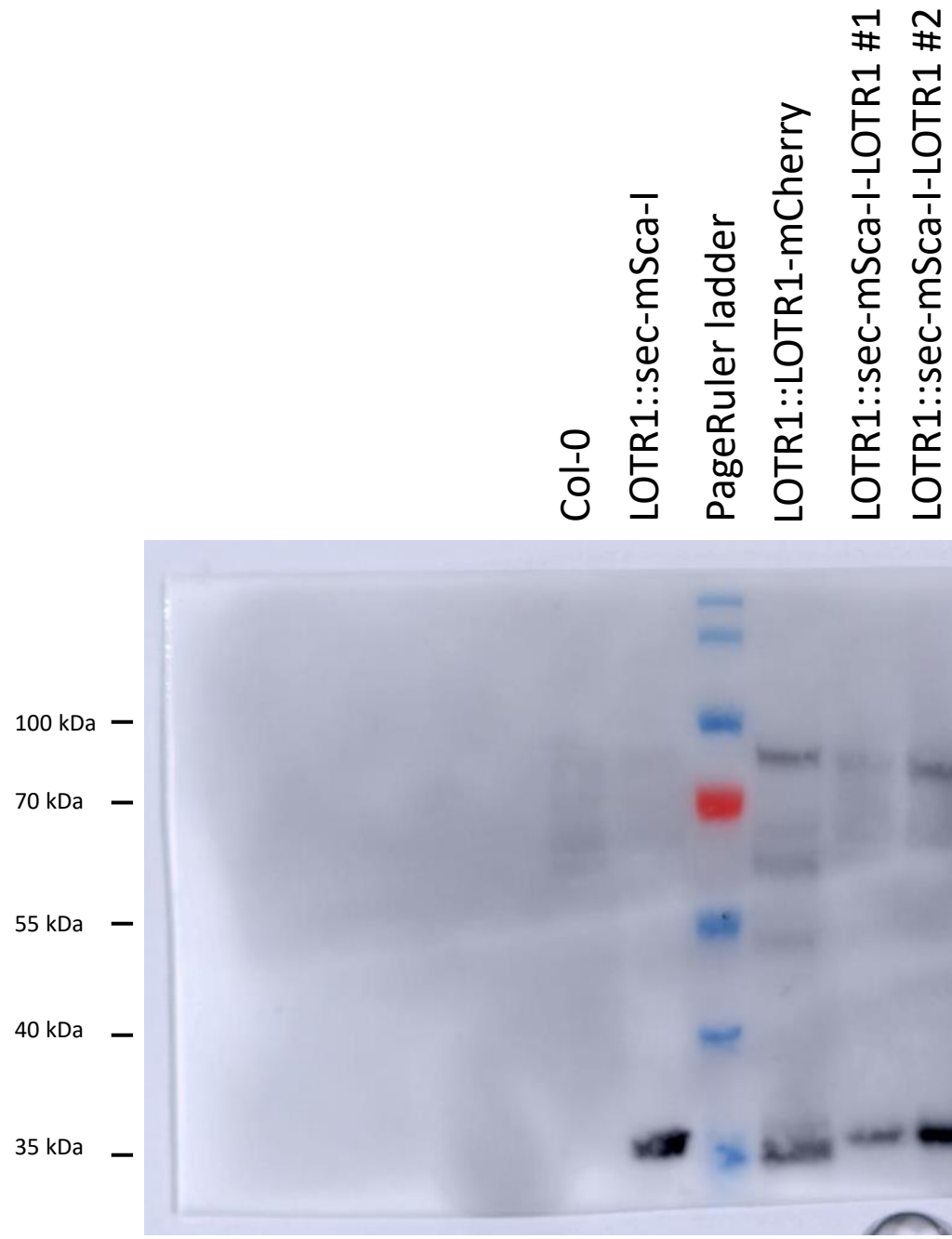

Supplement: Source data 1. [file elife-69602-supp1.zip › Kolbeck et al_source files/Figure 4-figure supplement 1-source data 1/Figure 4-figure supplement 1C-source data 1/20190404_LOTR1 Processing_Western Blot_Annotations.pdf]
